# Establishment and evaluation of a theater influenza monitoring platform

**DOI:** 10.1186/s40779-017-0144-3

**Published:** 2017-11-20

**Authors:** Jian Wang, Hui-Suo Yang, Bing Deng, Meng-Jing Shi, Xiang-Da Li, Qing-Gong Nian, Wen-Jing Song, Feng Bing, Qing-Feng Li

**Affiliations:** Center for Disease Prevention and Control of Beijing Military Region, 66th Heishitou Road, Shijingshan District, Beijing, 100042 China

**Keywords:** Influenza monitoring, Military, Establishment, Evaluation

## Abstract

**Background:**

Influenza is an acute respiratory infectious disease with a high incidence rate in the Chinese army, which directly disturbs military training and affects soldiers’ health. Influenza surveillance systems are widely used around the world and play an important role in influenza epidemic prevention and control.

**Methods:**

As a theater centers for disease prevention and control, we established an influenza monitoring platform (IMP) in 2014 to strengthen the monitoring of influenza-like illness and influenza virus infection. In this study, we introduced the constitution, influenza virus detection, and quality control for an IMP. The monitoring effect was also evaluated by comparing the monitoring data with data from national influenza surveillance systems. The experiences and problems associated with the platform also were summarized.

**Results:**

A theater IMP was established based on 3 levels of medical units, including monitoring sites, testing laboratories and a checking laboratory. A series of measures were taken to guarantee the quality of monitoring, such as technical training, a unified process, sufficient supervision and timely communication. The platform has run smoothly for 3 monitoring years to date. In the 2014–2015 and 2016–2017 monitoring years, sample amount coincided with that obtained from the National Influenza Surveillance program. In the 2015–2016 monitoring year, due to the strict prevention and control measures, an influenza epidemic peak was avoided in monitoring units, and the monitoring data did not coincide with that of the National Influenza Surveillance program. Several problems, including insufficient attention, unreasonable administrative intervention or subordination relationships, and the necessity of detection in monitoring sites were still observed.

**Conclusions:**

A theater IMP was established rationally and played a deserved role in the prevention and control of influenza. However, several problems remain to be solved.

## Background

The influenza virus has been responsible for influenza pandemics causing severe disruptions all around the world. In 1918, 1957 and 1968, the “Spanish influenza” A (H1N1), the “Asian influenza” A (H2N2) and the “Hong Kong influenza” A (H3N2) pandemics caused millions of deaths worldwide. Even in 2009, H1N1 influenza A led to between 200,000 and 400,000 deaths [1]. Influenza virus spreads through air droplet, and people in crowded places can be susceptible to infection, for example, in the military camps. In recent years, influenza has been a common infectious disease in the Chinese army, which directly disturbs military training and affects the soldiers’ health [2]. According to a research on the infectious diseases proportions in the Chinese army from 2001 to 2010, influenza virus-related respiratory infectious diseases headed the list of all of the infectious diseases, except in 2001, 2004 and 2005 [3]. Therefore, it is necessary and urgent to prevent and control influenza epidemics in the Chinese army.

An influenza surveillance system was used widely for influenza prevention and control [4–6], and virological data collected from laboratories or hospitals was a good indicator of influenza and provided a reasonable pre-peak warning at the regional level [7, 8]. China also established a national influenza surveillance (NIS) system, which covered 31 provinces. In 2014, as a theater for the Centers for Disease Prevention and Control (CDC), we established an influenza monitoring platform (IMP) to strengthen the monitoring of influenza-like illness and influenza virus infection. This paper investigates the establishment, operation, experiences and problems of a theater IMP; the IMP monitoring effect is also evaluated by comparing the monitoring data from the IMP with that of the NIS.

## Methods

### Constitution of influenza monitoring platform

Theater IMP is constituted of 3 levels of medical units, including monitoring sites, testing laboratories and a checking laboratory. The platform runs from October 1 to March 31 of the following year and covers the prevalent season of influenza. Monitoring sites are setup in basic units, mostly in regiment medical teams, which are responsible for collecting nasopharyngeal swab samples, transporting collected samples to the testing laboratory, and isolating and treating confirmed influenza patients. Nasopharyngeal swab and morbidity information are collected from soldiers or officers based on whether there is a fever combined with influenza-like symptoms. Testing laboratories are responsible for the reception, detection and storage of samples from monitoring sites, and they are set up in regional military hospitals. Between 3 and 6 monitoring sites typically are under the management of one testing laboratory. Testing laboratories also need to inform the monitoring sites of the detection results in a timely fashion in case the influenza viruses spread widely. A checking laboratory is a laboratory that runs a repeated detection on influenza virus positive samples from testing laboratories, which is set up in the theater CDC. The checking laboratory is also responsible for the disposal of influenza outbreak in regiments, training IMP staffs and supervising the platform.

### Influenza virus detection

Nasopharyngeal swabs are temporarily deposited in a virus culture medium and transported to the testing laboratory at a low temperature. The testing laboratory verifies the samples and morbidity information and later extracts nucleic acid from samples in a biosafety laboratory (BSL)-2 laboratory using commercial reagent kits. Nucleic acid is subsequently detected by real-time PCR using influenza A virus or influenza B virus specific primers. Usually, influenza A virus positive samples nucleic acid will be further detected for exact subtypes, such as H1N1 and seasonal H3. A reference gene, positive control and negative control are used to guarantee the quality of detection each time. The construction of the reaction system, the setting of reaction temperature and time follow the commercial kit instructions completely. All of the detection results are analyzed by a computer to minimize artificial error, including threshold baseline setting and Ct value determination. Monitoring sites receive the results once detection is completed. If there are influenza virus-positive samples, detailed information will be gathered to assess the risk of an epidemic outbreak. In this case, medical isolation and treatment would be performed immediately by medical teams at the monitoring sites.

### Quality control

We take steps to guarantee the quality of sample collection, transportation and detection, which is key to ensure efficient running of the IMP. First, staff are trained every year to ensure that all staff members are qualified for monitoring work; training content includes monitoring requirement, detection technique and biosafety requirements. Second, all of the consumables and reagents used in the IMP are uniformly purchased and issued to avoid the potential impact of different monitoring materials on sample collection and detection. Third, the supervision of monitoring sites and testing laboratories progress is an additional measure to understand the operational situation of monitoring work. Finally, all three levels of the units maintain timely communication on the detection results, morbidity information and preventive measures, by which we can determine and solve problems in a timely manner.

### Evaluation of the monitoring platforms

The checking laboratory obtains overall data from the IMP, including morbidity information regarding soldiers or officers with influenza-like symptoms, as well as the laboratories’ detection results of all of the samples. We compare our monitoring data with those from the NIS system to evaluate the platform’s capability of reflecting influenza epidemics in military camps. NIS data are collected from the website of the Chinese CDC (http://www.chinacdc.cn/). The comparison was determined by using Bivariate Correlation Analysis (SPSS V17.0); *P* ≤ 0.05 represents a statistically significant correlation.

## Results

### Basic situation of theater IMP

**Fig. 1:**
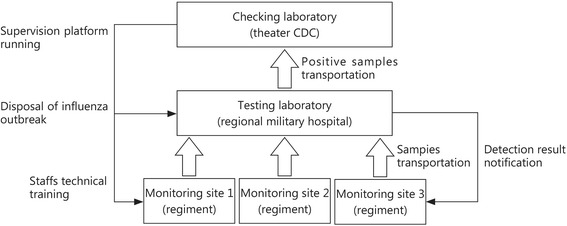
Constitution and procedure of the influenza monitoring platform. CDC. Center for disease prevention and control

The theater IMP has run for 3 monitoring years since October 2014 and plays a significant role in influenza prevention and control. We held 3 trainings for all staff from the monitoring sites and testing laboratories, which made it possible for them to accomplish their work in the IMP. Meanwhile, 3 supervisions were performed to understand, evaluate and improve the monitoring progress of monitoring sites and testing laboratories. The sample collection, transportation and detection were carried out smoothly during the 3 monitoring years, which implied that the IMP was well-designed, practical and feasible (Fig. 1).

### Primary monitoring data

As shown in Table 1, in the 2014–2015 monitoring year, seasonal H3 influenza A virus was the main influenza virus in the monitoring units, and the sample’s positive ratio was 17.92% for the whole year. In 2015–2016 monitoring year, influenza B virus was the main influenza virus, and the positive ratio was 19.26%, which was slightly higher than the first year. Seasonal H3 influenza A virus was the main influenza virus in the 2016–2017, and the positive ratio was 9.22%, which was lower than that observed in the first 2 years.

**Table 1:** Main monitoring data from the 3 monitoring years

Year	Samples collected (*n*)	Positive samples (*n* (%))	Influenza A virus subtype unknown (*n* (%))	H1N1 influenza A Virus (*n* (%))	Seasonal H3 influenza A Virus (*n* (%))	Influenza B virus (*n* (%))
2014–2015	1440	258(17.92)	79(30.62)	0(0.00)	170(65.89)	9(3.49)
2015–2016	1812	349(19.26)	48(13.71)	6(1.71)	31(8.86)	265(75.72)
2016–2017	1269	117 (9.22)	12(10.26)	0(0.00)	79(67.52)	26(22.22)
Total	4521	724 (16.01)	139(19.20)	6(5.13)	280(38.67)	300(41.44)

% represents the constituent ratio of the subtype samples in positive samples

### Time distribution of positive samples

As shown in Table 2, in the 2014–2015 monitoring year, the sampled influenza virus positive ratio was 39.77% in December 2014, which was the largest from all the months. In the 2015–2016 monitoring year, the sampled influenza virus positive ratio reached 36.26% in March 2016, which was the highest. In 2017, the positive ratio in January was 16.46%, which was the highest month.

**Table 2:** Time distribution of positive samples in the 3 monitoring years (*n*)

Year	October	November	December	January	February	March	Total
2014–2015							
Samples collected (*n*)	78	248	342	222	139	411	1440
Positive samples (*n* (%))	13 (16.67)	40 (16.13)	136 (39.77)	50 (22.52)	12 (8.63)	7(1.7)	258 (17.92)
2015–2016							
Samples collected (*n*)	104	181	246	473	455	353	1812
Positive samples (*n* (%))	4(3.85)	15(8.29)	0(0.00)	72 (15.22)	130 (28.57)	128 (36.26)	349 (19.26)
2016–2017							
Samples collected (*n*)	3	144	279	237	180	426	1269
Positive samples (*n* (%))	0(0.00)	18(12.5)	2(0.72)	39 (16.46)	12(6.67)	46(10.80)	117 (9.22)

% represents the positive ratio in monthly collected samples

### Monitoring data were coincident with NIS data

**Fig. 2:**
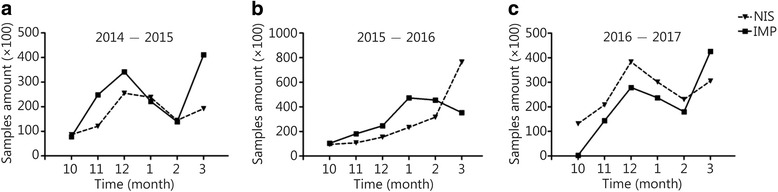
Time distribution of IMP samples’ comparison with NIS data, the sample amount time distribution comparison between IMP and the NIS in 2014–2015 (**a**), 2015–2016 (**b**) and 2016–2017 (**c**) monitoring year. NIS. National influenza surveillance; IMP. Influenza monitoring platform

To evaluate the platform’s capability of reflecting influenza virus activities in the monitoring areas, the monitoring data were compared carefully with that from the NIS system. As shown in Fig. 2a, the number of confirmed influenza patients in October 2014 was 8635, while the cases increased to a peak in December 2014 and later decreased to a trough in February 2015. In March 2015, the cases subsequently increased again. After bivariate analysis, the correlation coefficient *r* was 0.782, and the *P* value was 0.03, which implied that the IMP data were congruent with the NIS data. In the 2016–2017 monitoring year (Fig. 2c), the correlation coefficient *r* was 0.804 and the *P* value was 0.05, which meant the IMP data were also consistent with the NIS data. In the 2015–2016 monitoring year (Fig. 2b), however, we took great effort to prevent and control the influenza epidemic; Therefore, when the NIS data showed a peak in March 2016, the related IMP data did not demonstrate such a peak.

### Early-warning effect of influenza epidemics

According to the regulations of the Chinese PLA, three or more confirmed influenza patients occurring in a camp within a week is defined as aggregation of influenza epidemic (AIE). In the 3 monitoring years, 14 AIEs were confirmed in monitoring units. Seven AIEs were in the 2014–2015 monitoring year, 5 were in 2015–2016, and 2 were in 2016–2017. Among these AIEs, 3 occurred in military schools, 5 were in training bases, and 6 were in military camps. All of the AIEs occurred in late January or early February and involved 190 confirmed influenza patients. However, none of the aggregations spread, which implied that efficient monitoring was a useful measure to prevent and control influenza.

## Discussion

Influenza viruses primarily include influenza A virus and influenza B virus. Due to high variability, influenza A viruses, including H1N1, H5N1, H7N1, H7N2, H7N3, H7N7, H7N9, H9N2, and H10N8, are the typical culprits of pandemic influenza [9–12], while influenza B viruses only lead to limited influenza epidemics [13]. Influenza virus infection always leads to high fever, cough, runny nose and myalgia; many patients have severe pneumonia. Heart, kidney or other organ failure may cause death directly in the most serious cases.

The WHO has established a global influenza program (GIP) to monitor influenza case reports and carry out epidemiological analysis of human influenza, human avian influenza infection, human swine influenza infection and severe acute respiratory infection (SARI). GIP also promotes the development of and collaboration among, for instance, influenza laboratories, cessation of influenza pandemics, influenza vaccines production, monitoring data sharing, health education [14]. In the 2009 H1N1 influenza pandemic and 2013 Middle East respiratory syndrome (MERS) pandemic, GIP made great efforts in influenza prevention and control [15].

Influenza is a notifiable infectious disease in China. China also has a NIS system, which covers 31 provinces and contains nearly 1000 monitoring units. The China NIS system is a disease monitoring system with extensive coverage, is well-functioning, and represents a relatively large investment [16]. NIS has played non-negligible roles in influenza prevention and control in China.

Military camps have crowed populations, and military trainings expend soldiers’ physical and mental power, which makes the influenza virus easy to spread. This reality calls for an army IMP. We therefore established an IMP, aiming at early warnings for influence epidemics. Monitoring sites, testing laboratories and a checking laboratory were all designed and set up according to the actual operations of the Chinese army. The platform runs smoothly, effectively and played a deserved role in influenza prevention and control.

In all 3 monitoring years, the most common influenza virus were all consistent with that of NIS, which implied that IMP accurately reflected the epidemic status. As the IMP data of 2014–2015 and 2016–2017 monitoring years showed, the IMP epidemic peaks were congruent with the NIS peaks. Although the data from the 2015–2016 monitoring year was not consistent with NIS, this finding was attributed to the strict prevention measures implemented in early 2016. In the 3 monitoring years, none of the 14 confirmed AIEs spread widely, which implied that efficient monitoring was a useful measure to prevent and control influenza.

The main experiences could be summarized as follows: First, IMP made use of existing resources, such as setting up testing laboratories in regional military hospitals to avoid building redundant BSL-2 laboratories. Second, most of the monitoring units attached great importance to IMP; technical needs were met in a timely manner, and major problems were solved efficiently. Third, technical training was a significant measure to enhance staff and thus sample collection and detection quality; supervision was also an essential measure to maintain the IMP’s strong operations. Fourth, smooth communication, including detection results notification and emergency measures sharing, ensured a rapid response to epidemics.

Several limitations in this study should be noted. A portion of staff members at monitoring sites could not collect samples accurately and in a timely manner because they periodically placed an insufficient emphasis on the IMP work. Thorough and efficient health education or administration pressure may solve the problem in the future. Additionally, due to the army’s particularity, certain monitoring sites could not cover all soldiers in their regiments. Certain monitoring sites were distant from their testing laboratory, which made it difficult for the IMP staff to eliminate monitoring blind areas and transport samples in a timely manner. It may be possible to adjust distributions and affiliations between the testing laboratories and monitoring sites. Meanwhile, there would be an earlier warning of an influenza epidemic, leading to a lower risk of outbreak, if the virus’ nucleic acid could be detected more accurately and timely at the monitoring site. Therefore, more attention should be paid to the development of influenza rapid detection technology.

## Conclusions

In the past 3 years, a theater IMP has been established rationally and has played a deserved role in the prevention and control of influenza epidemics. However, several problems remain to be solved properly.

## Abbreviations

AIE: Aggregation of influenza epidemic
BSL: Biosafety laboratory
CDC: Center for disease prevention and control
GIP: Global influenza program
IMP: Influenza monitoring platform
MERS: Middle East respiratory syndrome
NIS: National influenza surveillance
SARI: Severe acute respiratory infection
